# Describing the safety of abortion at the population level using network-based survey approaches

**DOI:** 10.1186/s12978-022-01518-3

**Published:** 2022-12-27

**Authors:** Clémentine Rossier, Onikepe Owolabi, Seni Kouanda, Martin Bangha, Caron R. Kim, Bela Ganatra, Dennis Feehan, Casey Breen, Moussa Zan, Rachidatou Compaoré, Adama Baguiya, Ramatou Ouédraogo, Clement Oduor, Vincent Bagnoa, Sherine Athero

**Affiliations:** 1 Institute of Demography and Socioeconomics, University of Geneva, 40 Bd du Pont d’Arve, 1211 Geneva, Switzerland; 2 Institut National d’Etudes Démographiques, Paris, France; 3 Guttmacher Institute, New York, USA; 4 IRSS, Ouagadougou, Burkina Faso; 5 APHRC, Nairobi, Kenya; 6 UNDP/UNFPA/UNICEF/WHO/World Bank Special Programme of Research, Development and Research Training in Human Reproduction (HRP), Department of Sexual and Reproductive Health and Research, WHO, Geneva, Switzerland; 7 University of California, Berkeley, Berkeley USA

**Keywords:** Unsafe abortion, Abortion care seeking, Population-based data, Network-based survey methods, Confidantes' method, Anonymous third-party reporting method, Network scale-up method, Respondent-driven sampling, Health and demographic surveillance systems

## Abstract

**Background:**

Despite the negative impact of unsafe abortions on women's health and rights, the degree of abortion safety remains strikingly undocumented for a large share of abortions globally. Data on how women induce abortions (method, setting, provider) are central to the measurement of abortion safety. However, health-facility statistics and direct questioning in population surveys do not yield representative data on abortion care seeking pathways in settings where access to abortion services is highly restricted. Recent developments in survey methodologies to study stigmatized / illegal behaviour and hidden populations rely on the fact that such information circulates within social networks; however, such efforts have yet to give convincing results for unsafe abortions.

**Objective:**

This article presents the protocol of a study whose purpose is to apply and develop further two network-based methods to contribute to the generation of reliable population-level information on the safety of abortions in contexts where access to legal abortion services is highly restricted.

**Methods:**

This study plans to obtain population-level data on abortion care seeking in two Health and Demographic Surveillance Systems in urban Kenya and rural Burkina Faso by applying two methods: Anonymous Third-Party Reporting (ATPR) (also known as confidantes’ method) and Respondent Driven Sampling (RDS). We will conduct a mixed methods formative study to determine whether these network-based approaches are pertinent in the study contexts. The ATPR will be refined notably by incorporating elements of the Network Scale-Up Method (NSUM) to correct or account for certain of its biases (transmission, barrier, social desirability, selection). The RDS will provide reliable alternative estimates of abortion safety if large samples and equilibrium can be reached; an RDS multiplex variant (also including social referents) will be tested.

**Discussion:**

This study aims at documenting abortion safety in two local sites using ATPR and RDS. If successful, it will provide data on the safety profiles of abortion seekers across sociodemographic categories in two contrasted settings in sub-Saharan Africa. It will advance the formative research needed to determine whether ATPR and RDS are applicable or not in a given context. It will improve the questionnaire and correcting factors for the ATPR, improve the capacity of RDS to produce quasi-representative data on abortion safety, and advance the validation of both methods.

## Plain English summary

Representative data on how women induce abortions and their consequences are central to measurements of abortion safety. However, due to the stigmatized nature of abortion, measuring the details of the process is challenging when the latter occur out of the realm of the law and do not result in complications registered in hospital statistics. Hence, there is sparse empirical population-level data on how women terminate their pregnancies in countries where access to abortion services is highly restricted, as well as little data on the side effects and complications associated with the methods they chose and health seeking for these complications. Recent developments in indirect survey methodologies to study stigmatized/illegal behaviour and hidden populations are likely to improve the quality of data collected on abortion safety in restrictive contexts: all are based on the sharing of information on stigmatized practices in social networks. We propose to refine and pilot two such network-based methods to validate their use for collecting (quasi) representative data on abortion safety in large population health surveys. These two approaches are: (i) a modified Anonymous Third-Party Reporting method (ATPR) integrating elements of the Network-Scale-up Method (NSUM) and (ii) Respondent-Driven Sampling (RDS). We will conduct this study in two African Health and Demographic Surveillance Systems (HDSS) sites, one urban (Nairobi, Kenya), and one comprising a town and adjacent villages (Kaya, Burkina Faso).

## Background

This article presents the protocol of a study aimed at improving the measurement of the safety of abortion in countries where access to safe abortion services is restricted, by applying and developing two network-based approaches. New measurement methods are needed because information on the abortion process itself have become central to the understanding of unsafe abortion. The circumstances under which women obtain induced abortions have indeed changed rapidly in the last 25 years. The most influential change has been the use of medical abortion in many contexts where access to legal abortion services is restricted [1, 2], accompanied by a growing use of medical vacuum aspiration for surgical termination and improved health systems in some contexts, for example in Honduras or Pakistan [3, 4]. These evolutions have rendered many illegal abortions safer and some legal abortions unsafe (dilatation and curettage): at the same time, unsafe abortions still take many lives around the globe, especially where health systems are weak [5].

For these reasons, the World Health Organization (WHO) has moved away from operationalizing the definition of abortion safety dichotomously and based on legality (legal-safe, illegal-unsafe) and has proposed that abortion safety is measured along a continuum and according to the specific circumstances of the abortion—namely the skills of the person providing the abortion and method which both have to comply with minimal medical standards- which, taken together, define abortions as safe, less safe or least safe, according to current WHO guidelines. The share of safe/less safe/least safe abortions in a setting with no information can be modelled from empirical data on methods and providers in other settings [6, 7]; however, these models need to rely on as much direct evidence as possible. The WHO also stresses that the morbidity resulting from abortions at different levels of safety need to be better measured, as abortion mortality has declined.

Data on how women induce abortions (method, setting, provider) and their consequences (post abortion symptoms and care seeking) have thus become central to estimate the safety of abortion. In contexts where access to legal abortion services is highly restricted, collecting such data from informal abortion providers is (usually) impossible; at the same time, measuring the details of abortion procedures at the population-level through self-report in surveys remains challenging due to the stigmatized nature of abortion. Indeed, such direct questions do yield close to no response in many restrictive contexts [8], while approaches that aim to enhance the privacy of survey respondents such as ACASI typically do not improve response rates for abortion dramatically, or allow only for one question to be asked in the case of the List experiment [9]. Hence, there is sparse empirical population-level data on how women terminate their pregnancies in restrictive contexts, as well as little data on complications associated with the methods they chose and health seeking behaviours, as shown by a recent systematic review of population-level data on the safety of abortions [7].

Recent developments in survey methodologies to study stigmatized/illegal behaviour and hidden populations^1^ are likely to improve the quality of data collected on abortion care seeking pathway (method, provider, setting) in contexts where access to legal abortion services is highly restricted. These methods all rely on the fact that information about the stigmatized behaviour circulates within social networks. In this study, we propose to apply and improve on two such network-based methods to document abortion safety^2^ in two sites, one in rural Burkina Faso and one in urban Kenya. In what follows we will discuss the literature on these two methods: the Anonymous Third Party Reporting (ATPR) method—and Respondent Driven Sampling. We will also discuss a third method, the Network Scale Up Method (NSUM), because some of its features can be used to improve on the ATPR (Table 1).

**Table 1:** Comparing the characteristics of the ATPR, NSUM and RDS

	ATPR (applied in this study)	NSUM	RDS (applied in this study)
Results	Counts and detailed information about abortion cases	Counts of abortions cases	Detailed information about abortion cases, no counts
Recruitment and questionnaire	Respondents in a random sample survey report anonymously on abortions occurring to close female relations 15–49, listed using a network generating question	Respondents in a random sample survey try to estimate the number of abortions which occurred in their entire network of acquaintances, whose size is estimated	A set of initial respondents (seeds) report on their own abortion and recruit other abortion seekers through their networks
Precision of data collected	Information on a set of finite close relations: information still relatively accurate	Not possible to ask for details on each abortion	Direct information from the abortion seekers: highest precision but still non-clinical knowledge
Assumptions	Respondent report accurately about abortions of friends (no social desirability bias) Respondents know about friends' abortions (no transmission bias) Abortion seekers have the same networks than general population (no barrier bias) Friends have the same networks than respondents (no selection bias) The network generating question may be biased towards friends with abortions	Respondent report accurately about abortions of entire network (no social desirability bias) Respondents know about abortions in entire network (no transmission bias) Abortion seekers have the same networks than general population (no barrier bias)	Abortion seekers self-disclose in an interview setting whose privacy is guaranteed by a network member (no social desirability bias) Abortion which constitute membership in the population must create connections amongst the members of the population, members must share active ties, and abortion is objectively verifiable (no transmission bias)
Correcting for biases	Transmission, barrier and selection biases have been corrected for in an ad hoc fashion so far. The social desirability bias cannot be corrected for	A model has been developed to correct for transmission and barrier biases; the social desirability bias cannot be corrected for but is acknowledged.*	If equilibrium is reached (if the characteristics of new recruits are independent of those of the abortion seeker who recruited initially), then assumptions are met and the sample is unbiased
Representativity of data collected	Theoretically, quasi representative sample of abortion seekers, if biases are inexistent or corrected for	Theoretically, quasi representative count of abortions, if biases are inexistent or corrected for. But estimates are very sensitive to corrections	Theoretically, representative sample of abortion seekers if characteristics of new recruits are independent of those of the seed (i.e. if equilibrium is reached)
Internal validation	Internal validity check using known quantities	Internal validity check using known quantities	
Level of application	Can be applied to all levels: local to national	Can be applied to all levels: local to national	Local
Effort needed	Easy to standardize across times and locations: a module in a population survey	Easy to standardize across times and locations: a module in a population survey	Need for good local knowledge to locate seeds; time and effort intensive

^a^This feature of the NSUM will be used to improve on the ATPR

### The Anonymous Third-Party Reporting (ATPR) method (or confidantes' method)

ATPR was developed to collect quantitative data on induced abortion in restrictive contexts, by asking survey respondents to report anonymously on the recent abortions which occurred to a set of close female relations previously listed, including details on the abortion procedure and the complications experienced. The ATPR method is based on ethnographic work in Burkina Faso showing that information on abortions circulates in social networks in spite of this practice being strongly stigmatized, because women and couples cannot access abortion services otherwise [11]: information sharing is restricted to close relations and to people who can help in accessing abortion services, i.e. other abortion seekers or abortions providers. The ATPR defined close relations as people with whom respondents exchange private (i.e. secret, intimate) information; only female relations aged 15–49 (the demographic reproductive age group)—who can have abortions- are listed. Whilst the first tests of this method in Burkina Faso and Zambia and recent applications in PMA2020 surveys have yielded plausible results [12–14], four limitations have persisted. (i) First, different biases can affect ATPR results: *transmission bias -*respondents sometimes do not know about the abortions of their close relations-, *barrier bias* -abortion seekers' social networks may differ from that of average women- and *selection bias* -close female relations may have different networks than the general population-Up until now, these biases have been corrected for in an unsystematic way: different propositions coexist [14–19]. (ii) Second, it remains unclear in which contexts these biases are low and ATPR is applicable and in which contexts it is not the case [15, 16]. The existence of a transmission bias can be ascertained a priori, through a qualitative study documenting whether women with unintended pregnancies talk to close network members to locate underground abortion services [20]; but selection and barrier bias can be detected only when applying the method, suggesting the need for a quantitative pre-test. A fourth bias -*social desirability-* (not wanting to report on network member abortion or in the opposite overreporting such events) [15, 21] could also be probed in a pre-test. (iii) Third, the ATPR results ideally need to be validated, internally or externally. (iv) Fourth, the best standardized and unbiased network-generating question remains to be identified, as mentions of abortion in the presentation of the survey or in the network-generating question itself has been shown to inflate the estimates, and as a question focused on “secret sharing” may -perhaps- inflate the responses [18].^3^

### The Network Scale-Up Method (NSUM)

Recent advancements in another third-party reporting method called the Network Scale-Up Method (NSUM) could provide viable solutions to the first limitation. The NSUM has been used in recent years to estimate the size of epidemiologically hidden and stigmatized populations in HIV research (e.g. sex workers, men who have sex with men, injecting drug users) [26, 27], and recently to estimate the number of abortions [28, 29]. The estimates of the NSUM are generated by collecting information on two elements from a representative sample of respondents: (i) how many members of the hidden population they know, and (ii) *estimates* of the overall size of their social networks. This data is then aggregated as a ratio to generate population level estimates of the size of these groups. The overall network size of respondents (often defined as all people known by name, contacted in the last 2 years, and who could be contacted if needed) is estimated by asking respondents about the size of circa 20 *known* populations (i.e. number of people named Smith etc.). Since the ratio of Smith to the entire population is known, the number of people known to a respondent can be deduced from the number of Smith’s she knows. Over the years, much attention has been paid to the biases of the NSUM which can be corrected, namely the *transmission* (respondent not knowing about the practices of interest in their networks) and *barrier* effects (hidden populations having different networks compared to the general population). In other words, when stigmatized practices are not well known to others or when individuals with the stigmatized practice do not interact with the general population, the NSUM estimates will be heavily underestimated as is obvious when the NSUM was applied to abortion in Iran [28]. The NSUM also underlines the existence of a third bias, *social desirability*, which can affect the estimate but cannot be corrected for [21]. Recently, a generalized NSUM formula has been proposed where the two first biases, measured directly from members of the hidden group (for example using self-report or the Respondent-Driven Sampling technique), are used to correct the NSUM estimates [21]. But estimates remain very sensitive to small differences in the different measure; a recent application to abortion yielded implausible results even after corrections [29].

In their 2016 publication [30] Feehan et al. report on the impact of asking respondents for information about a smaller network of relations (circa 100 people you ate a meal with, instead of circa 250 people you know), which bettered the quality of the information collected. The NSUM is therefore starting to go in the direction of the ATPR, which requests information about an intimate network of women. However, because close relations are a subset of all relations, *selection biases* will additionally need to be considered in the ATPR, as well as the independence of the network generating question versus the practice of interest. Nevertheless, ATPR has several advantages over the NSUM: only counts can be obtained with the NSUM, while details on the procedures can be collected via the ATPR. Second, the transmission bias is obviously much weaker in networks of close relations as opposed to circles of people you "know" or "ate a meal with". Accordingly, plausible data on abortion have been collected via ATPR in some places, while NSUM failed to do so. In this study we will thus apply the ATPR rather than NSUM. However, some features of NSUM can be used to improve on the ATPR, especially its systematic formula to correct for transmission and barrier biases and its attention to the issue of social desirability.

### Respondent driven sampling

The Respondent-Driven Sampling (RDS) is another network-based approach [31–33] which could measure the safety characteristics of abortion with some degree of robustness. The RDS works through a peer recruitment design. In RDS studies, seeds (members of the population of interest) are recruited and, using a fixed coupon system which enables the tracking of the recruitment process, go on to recruit other participants who then recruit others in a continuous chain till equilibrium and saturation is reached. Equilibrium is the point at which the characteristics of the sample are independent of the seeds and do not change significantly regardless of how many more people are recruited. Theoretically, the sample population obtained is thus representative of the entire (albeit local) population with the characteristic of interest, as are the quantities measured within it. Another key advantage of the RDS for abortion safety is that because respondents have experienced the event of interest, the quality of information on the process of abortions is likely to be better than data collected via third party reporting. The RDS has successfully produced quasi-representative samples of hidden populations in a vast diversity of settings and for a diversity of stigmatized practices in over 100 published studies [31–33], including for women who aborted following a war violence induced pregnancy in the DRC [32]. An RDS for abortion was also recently implemented with great success in Zanzibar [34]. However, in the last two studies, sample sizes were small and equilibrium was not checked.

To implement an RDS, a number of assumptions have to be met, including that the activities which constitute membership in the population (in this case abortion) must create connections amongst the members of the population, that the members must share active ties, and that the trait defining the population is objectively verifiable [10]. When these assumptions are met, there is no transmission bias. To verify the extent to which these assumptions are met in the case of abortion, we conducted in 2018 a systematic review of the literature on information sharing by abortion seekers in low and middle-income countries since 2000, which generated 79 studies for inclusion [20]. An analysis of these articles shows that disclosure to network members (beyond the partner and family) depends on the context of abortion service provision and on the level of stigma. Where women can access abortions (whether legal or not) *anonymously* (through ads, flyers, internet, etc.), abortion seekers do not need to disclose beyond their family members, and do not if stigma is strong. For example, qualitative interviews with unmarried women in Ethiopia showed that they managed to access (legal and illegal) services without revealing their situation to anyone they knew [35]; same for legal service users in Colombia [36]. On the other hand, in contexts where abortion services (whether legal or not) *cannot be accessed anonymously*, women (or their family members) *have* to reach out to members of their network -especially recent abortion seekers- to locate an abortion provider or method. Abortion stigma, while often high, is dealt with by disclosing only to members of the network bound to secrecy. The vast majority of sites described in the systematic review, and this in all the world regions (Africa, Asia, Latin America), fell in this category. For instance, in Saudi Arabia, 82% of self-reported misoprostol users in a survey knew another misoprostol user [37]; in Brazil, 1 year after the abortion, 70% of teenage post-abortion care users reported knowing a friend who had terminated [38]; an illegal abortion provider in a qualitative study in Kenya confirmed that prior clients were the major source of new clients [39]; etc. Moreover, the review reveals that the diffusion of medical abortion does not seem to make women less dependent on their network, because information on medical abortion is not available anonymously and publicly in these low and middle-income contexts. The existing literature thus confirms that the assumptions of the RDS (no transmission bias) are met for a large majority of recent abortion seekers (in the order of magnitude of 7 or 8 abortion seekers out of 10), *but only in contexts where abortion services cannot be accessed anonymously*.^4^ In fact, one RDS experiment in South Africa, where abortion services even in the informal sector are advertised on flyers, posters etc., seemed less successful as it yielded only 43 cases of women who had an informal sector abortion, a third of which were female sex workers [40].

Over the years, methodological improvements have been developed regarding how RDS studies are designed, data is collected and statistical estimates are generated. These developments include managing the flow of participants into the study by utilizing a systematic coupon reduction process which is the reduction of the number of coupons to be distributed (from 3 to 2 and then from 2 to 1) at some specific waves [31, 33]. A formative study is also deemed necessary to gain an in-depth understanding of local dynamics in terms of social relations and stigma, and to design the practical implementation modalities (seed selection, type of compensation, place of interviews, etc.) A bootstrap approach has commonly been employed in RDS, to generate confidence intervals around estimates as these are more appropriate for the complex sampling underlying this method and avoid too narrow confidence intervals [32]. To take into account the fact that active ties are maintained not necessarily with other abortion seekers but sometimes with friends of abortion seekers, we developed during the preparatory phases for the study-and tested through modelling- a *multiplex RDS design*. This RDS innovates by occasionally involving the member of another population in the referral chains [41], i.e. by asking respondents to recruit another abortion seeker (as in the classic RDS) *or* a person who is close to one or more abortion seekers.^5^

Compared to the ATPR, the RDS has an evident advantage: a sample of abortion seekers where equilibrium has been reached is in principle quasi-representative. Therefore, the data obtained through RDS can be used to estimate the size of the transmission and barrier bias for the ATPR, and are probably more reliable than correcting factors obtained from self-report. The results obtained through ATPR-before and after correction—can also be compared to those obtained through RDS. The formative study (qualitative and quantitative) done in RDS could also usefully be applied to the ATPR. On the other hand, RDS could integrate features inspired by the ATPR, like targeting a large sample of abortion seekers, and using social referents to reach abortion seekers. But the RDS can only be applied in localized sites, while the ATPR can be used at all levels.

## Purpose and aims

The purpose of this study is to contribute to the generation of reliable population-level information on the safety of abortions in contexts where access to legal abortion services is highly restricted. To achieve this purpose, we will pursue two aims in the present study: first, generate reliable *local* population-level data on abortion safety in two sites by applying two network-based methods, ATPR and RDS; second, improve our capacity to collect such data at a *larger scale* in the future in similar settings by improving on these two network-based methods and comparing their results.

### Methods

To attain these two aims, we propose to use the following methodological strategy:

1. We will obtain population-level data on abortion care seeking (method, provider, setting and post-abortion symptoms and care-seeking) in two sites in Kenya and Burkina Faso where access to legal abortion services is highly restricted, by applying Anonymous Third Party Reporting (ATPR) (also known as confidantes’ method) and Respondent-Driven Sampling, both modified with features that can potentially improve their implementation or accuracy.
2. We will modify the Anonymous Third Party Reporting (ATPR) method by refining and validating the method for future use in large scale surveys by: 2a. developing a formative study to determine whether the ATPR is pertinent in that context; 2b. identifying the best network-generating question; 2c. measuring the size of the two biases (barrier and transmission) which affects its estimates using data generated by RDS and self-report, and if pertinent, correct the ATPR estimates using the generalized Network Scale-Up Method (NSUM) model, correct also for selection bias; 2d. assessing the potential for self-report data and stigma data to help in yielding ATPR bias estimates in different contexts; 2d. conducting internal validation for the ATPR using known quantities; 2e. comparing ATPR safety estimates with those from RDS.
3. We plan to improve on RDS for abortion by 3a. testing a multiplex design; 3b. obtaining a large sample of abortion seekers and reaching equilibrium.

### Study setting and population

The study will be conducted in two Health and Demographic Surveillance Systems (HDSS), the Nairobi HDSS in Kenya [42] and the Kaya HDSS in Burkina Faso [43]. In both countries, induced abortion is allowed on narrow grounds. In Burkina Faso, abortion is allowed on the grounds of rape, incest, fetal impairment, saving the woman’s life and health. In Kenya, abortion is legal if it is performed to protect the women’s health or if the pregnancy is life-threatening or the pregnant women’s life is otherwise endangered.

HDSS track a population and its vital events (e.g. births, deaths, migration) in a given catchment area, which often corresponds to a health district [42–44]. Once a surveillance area has been delineated, a census is undertaken; fieldworkers then visit each household at least once a year to update the household roosters. They document all in-migrations, out-migrations, deaths and births which occurred in the households since the last round. HDSS have been especially useful to study causes of deaths [44]; they also host clinical trials, and epidemiological or demographic studies.

HDSS offers several advantages to study abortion safety at the population level in contexts where access to abortion services is highly restricted: (1) the RDS needs to be applied at a local scale; (2) these two HDSS are run by Human Reproduction Programme Long term Institutional Development Hubs (HRP LID—HUBS) in sub-Saharan Africa (African Population Health Research Center, APHRC, in Kenya and Institut de Recherche en Sciences de la Santé, IRSS, in Burkina Faso), two institutions which have high research and dissemination capacities (both research dissemination and institutional experience with community-based work), key aspects for an innovative project on a sensitive topic. This choice allows us to strengthen research capacity, with the aim of having HRP LID-HUBS researchers using, advancing and disseminating state-of-the art network-based survey methods for sensitive behaviours and abortion research in other highly restrictive settings.

The systematic review [20] confirms that both sites meet the criteria for network-based approaches (abortion services cannot be accessed anonymously: low transmission bias among close relations). These two sites also represent some of the diversity of sub-Saharan Africa (from rural to small town in Kaya to large city in Nairobi, in West and East Africa). Validating network-based approaches in rural, small town and large city settings is a necessity, as dynamics of social relations and disclosure are likely to vary across types of residence.

### Overall study design: two phases

The two HDSS sites will obtain approvals from their local institutional review boards prior to the start of the project. The project is divided into two phases (Table 2). In a first phase, each HDSS will conduct a formative research, where the existence of the transmission bias will be probed in a qualitative study and the other biases will be investigated by conducting a quantitative pre-test for the ATPR and for the RDS. The study will then move to phase 2 (main survey: ATPR and RDS) if the data collected during phase 1 shows that the ATPR and RDS can be implemented for abortion in that site. For RDS, this means that phase 1 can produce at least 6 seeds that can successfully recruit 30 respondents. For ATPR this means that abortion rate obtained fall within in a plausible range.

**Table 2:** Study design

	Within HDSS	Outside HDSS	
Phase 1: Formative research	Qualitative study n = 20 in-depth interviews	Pre-test ATPR n = 100	Pre-test RDS n = 30
Phase 2: Main survey	ATPR n = 2000 Cross-sectional random survey collecting retrospective information (last 3 years) on abortion of close relations and on respondents' abortions		
	RDS n = 500 Prospective data collection with women who had abortion last 3 years (or individuals close to an abortion seeker)		

#### Phase 1: Formative study: qualitative study and pre-tests for ATPR and RDS

In phase 1, each HDSS will conduct a qualitative study (in the HDSS area) and pre-test the ATPR and RDS instruments and procedures in two different locations both outside of the HDSS area.

##### Qualitative study

The qualitative study will produce contextual knowledge on the practice of abortion, the circulation of information on abortion and abortion stigma, with the aim of testing whether the basic condition for ATPR / RDS use for abortion (low transmission bias among close relations) is met. The qualitative study could in addition provide a number of seeds for the main RDS survey. The qualitative material will also attune us to local terms used for abortion, diversity of methods used locally, etc., which will help ensure the translations of the questionnaires are correct and that response categories are pertinent. To this end, 20 in-depth interviews (IDI) will be conducted with a diversity of women living in the area willing to talk about women’s health problems, and referred by key informants in the community. This low number was deemed enough as several qualitative studies on the same topic have already been conducted in these countries showing that transmission bias for abortion is low among close relations; these IDI are thus confirmatory for the study sites. Informed consent will be sought and privacy ensured. The topics to be discussed in the IDI will include: (i) features of close female networks; (ii) the difficulties involved in deciding what to do when faced with an unplanned pregnancy, accessing abortion services, dealing with complications; how networks are involved in these various steps; (iii) social perceptions of and stigma surrounding abortion. The exact direction and content of discussions will be determined by participants. IDI will be recorded, translated (in French or English) and transcribed.

##### Pre-tests of the ATPR and RDS

In a location outside of the HDSS area (but similar in features), the questionnaire for the ATPR will be tested on 100 women aged 15–49. The questionnaire will include two successive network generator questions (the internationally validated “people you have discussed important matters with during the past year” as well as “up to three women you share secrets with”). The test of ATPR will advance knowledge on the network generating question best suited to investigate abortion and verify that the different biases (transmission, barrier, selection, social desirability) do not preclude the collection of data on abortion. The RDS pre-test will be conducted in a second location, also outside of the HDSS area, but also similar. The aim of the pre-test for the RDS is to fine-tune the RDS procedures (seeds, location for the interviews, etc.) and ascertain that abortion seekers in the study context accept to conduct interviews following peer-referral and to disclose their abortion in that context. Women recruited from 6 seeds following the RDS procedure (see more below) will be interviewed (up to a maximum of 30 individuals or 2 months of efforts). Among the six, three multiplex seed will be tested, in which respondents will be asked not only to recruit another abortion seeker but can also recruit somebody who knows an abortion seeker.

#### Phase 2: Main survey

In each site, the two components of the study will be conducted one after the other in the HDSS area. The ATPR will be run first and respondents will be warned about the coming RDS survey; they will be offered a coupon, to give the relation or fieldworker in case they are approached for the RDS. The RDS will start as soon as the ATPR stops. In the Kaya HDSS, three sub-geographies will be considered, distinguishing the rural part under surveillance from the urban part itself comprising of both formal and informal settlements. The goal is to make sure the ATPR sample is representative of all sub-geographies (that the sampling frame is complete for all areas, which necessitates extra work for informal areas) and that RDS seeds are diversified from that point of view.

### Data collection: Anonymous Third Party Reporting method

#### Sampling, inclusion criteria, and interview setting

We will conduct a cross sectional representative household-based survey among women aged 15–49. We will draw a simple random sample from the sampling frame of women 15–49 usually residing in the HDSS and randomization tables. We will use Excel to generate random numbers that will be apply to the ordered list of women in the area. The definition of residence may vary slightly across sites. To calculate sample size, we used estimates of abortion rates from published numbers in national studies or global estimates; numbers indicate a low 22 abortions per 1000 women aged 15–49 in rural Burkina Faso to a high of 48 abortions for 1000 15–49 in Kenya. The number of women aged 15–49 living in the HDSS area varies from 8500 in Nairobi to 10,000 in Kaya. Using these inputs and a random sample design, we determined that 1500 women in each site were enough to yield reliable abortion safety estimates across the two sites, assuming a reporting rate of 50% (one in two women knowing about the abortion of their close relation will report it in the survey) and two close relations. Along these assumptions, we will collect data on about 150 abortions in Kaya, and the double in Nairobi. To anticipate on possibly lower reporting rates and number of relations or abortion rates, we increase the sample size to 2000 in each site. All women^6^ who consent to participate in the study will be interviewed using the self-report/ATPR tool, in a private setting in their home or close surroundings, face to face by an interviewer recording answers on tablets.

##### Questionnaire

The questionnaire will include five sections.

- Sociodemographic characteristics and reproductive history of the respondent (including questions on her own abortions, towards the end of the interview).
- List of close network members, only females aged 15–49, with a standard network generator question from international social surveys and the shared secret question. Special care will be given to train fieldworkers and monitor them when administering this section, as interviewer effects have been identified in the literature for network generating questions.
- Characteristics of network members: sociodemographic characteristics (including whether in the area or not, since when in the area and since when a close relation), reproductive history, number and timing of any induced abortions in the past 3 years, how the abortion was induced (provider, setting, method), gestational age, post-abortion complications, treatment obtained for post-abortion complications, abortion related stigma, number of close relations by confidants. To maintain confidentiality, we will not collect information on relations' names but will ask them to provide nicknames or fictional names for each.
- Measures of abortion related stigma at the community level, personal level and internalized.

### Data collection: respondent-driven sampling

#### Recruitment, inclusion criteria and interview setting

When the ATPR is done, a different team of fieldworkers will start the RDS. To begin the RDS sample in the HDSS area, following the tested procedures, we will purposively select a sample of 8 to 12 women who reside in the area and have had abortions in the last 3 years as seeds to recruit our target population; they will be selected to represent the demographic, economic, residential and occupational diversity of the sites (see section on ethics in the discussion for details on seed recruitment). After providing informed consent, each seed will be interviewed face-to-face and provided with 3 coupons to recruit their peers who should meet the same inclusion criteria (reside in the site and have had an abortion, or in a multiplex design, reside in the site, and be close to someone who has had an abortion; pilot results will determine in which circumstances to recruit a social referent). Three coupons are given based on previous studies to avoid recruitment chains dying off at the beginning due to attrition. Coupons will be coded with a unique ID number so that recruiters can be linked to their recruits. They will also contain a phone number to call with enquiries, activation and expiry date. The research assistant will be allowed to join the respondent at the place the latter finds convenient for the interview; the interviewer will make sure the proposed place meets all confidentiality and privacy criteria before starting.

As in all RDS studies, each participant will receive two compensations (to be determined in accordance with local practices and national ethics committees, i.e. phone credits and/or travel expenses) for each successfully recruited additional participant. According to Heckathorn 1997, these rewards are more symbolic than substantive with studies showing that secondary compensation is particularly effective in facilitating the peer/social pressure needed to secure compliance to participate in studies within RDS networks. When participants return to the field office to receive their secondary compensation for recruiting participants, they will complete a questionnaire addressing if they tried to give coupons to other women who refused to accept it and why they were declined. All new recruits will be screened for eligibility to participate in the study after which informed consent will be taken and the questionnaire administered. They will then be trained to recruit other women and provided with coupons. The sample sizes of the RDS will be determined either during fieldwork, if saturation is reached and new respondents' characteristics are independent of those of the initial seed, or when the sample reaches a maximum of 500.

##### Questionnaire

The questionnaire administered to RDS participants will include:

- Sociodemographic characteristics and reproductive history of the respondent.
- Abortion history including number and timing of any induced abortions, how the abortion was induced (provider, setting, method), gestational age, post-abortion complications, treatment obtained, stigma (to be skipped for non-abortion seekers in multiplex RDS)
- Questions to estimate the biases of the ATPR: apply the ATPR modules to count close relations, characteristics of close relations, whether they had abortions, whether they told the respondents, how it was induced and any medical sequelae. For abortion seekers the module also contains the following questions: whether these close relations know about their abortions (whether respondents told them directly, respondents suspect they know even when not told).
- Stigma: three stigma scales will be used: community level, personal attitudes and internalized.

### Data management

All data collection in the main survey will be conducted on handheld android tablets using the mobile data collection software SurveyCTO collect. SurveyCTO is a platform that facilitates offline electronic data collection. Electronic data collection will be used to allow for increased security of collected data; tablets will be password-protected and completed consent forms and surveys will be uploaded to a secure server at the end of each day. Only the research team (University of Geneva and HDSS sites) will have access to the server. University of Geneva researchers will set up the secure server for the study and provide access to each site for the duration of the study.

### Plans for analyses

The analyses will focus on the two network methods applied to measure abortion safety (RDS and ATPR). The ATPR and RDS will provide data on what methods women use to have an abortion, where they have abortions and with what kind of provider, what side effects and post abortion care seeking behaviours they report.

The *RDS data* will be analyzed using the Respondent-Driven sampling analysis tool (RDSAT) or alternative tools in Stata or R, modified to take into account the multiplex structure if needed. For each site, we will examine recruitment patterns amongst respondents, and generate both crude point estimates for the sample recruited and population adjusted estimates of variables of interest including: women’s demographic characteristics, the proportion of women obtaining abortions from different types of providers, those experiencing complications, those seeking care etc. Additional analyses may include multivariable analyses examining the relationship between abortions with various degrees of safety or care seeking for complications and sociodemographic variables. We will also estimate the transmission rate using information on the number of respondents' close relations and the number who know about her induced abortions and its sequelae. We will measure the barrier bias by comparing the network sizes/structure of RDS respondents to that of all women in the ATPR survey.

Using the *ATPR data* and for each site, we will describe women’s demographic characteristics and that of their network of close relations. We will check which network-generating question yields the less selected sample of friends, and whether one question yields a sample that contains more abortion seekers, at constant characteristics. We will correct for selection bias. We will estimate the proportion of close relations obtaining abortions with different methods, different types of providers, those experiencing complications, those seeking care etc. We will do the same for women' self-reported abortions. For the ATPR point estimates, uncertainty intervals will be generated using a bootstrap or a pooled sample approach to account for the non-independence of the sample of women reported on [17]. Using the estimated transmission and barriers rates through the RDS (and perhaps self-report) and the generalized NSUM model, we will correct the ATPR estimates of the abortion rate if pertinent. We will analyze the correcting factors by socioeconomic level and stigma, to further our capacity to generalize these correction factors. We will further validate the ATPR estimates by applying ATPR to indicators drawn from the reproductive history collected for close relations and respondents (contraception), and by comparing the ATPR estimates to those from national estimates.

## Discussion

Altogether, this study will further our knowledge on abortion safety by allowing the collection of relatively representative data on a large number of recent induced abortions in two contrasted sites in sub-Saharan Africa, one in a rural and small-town setting in West Africa and one in the slums of a capital city in East Africa. Sociodemographic and safety characteristics of abortions across the two sites may vary widely, thus enlarging the scarce empirical-base on this topic in the region.

Also, this study will advance our knowledge of the two network-based methods applied and refined to measure abortion safety. While ATPR has now been used in a diversity of settings with success (although using ad hoc corrections procedures), this method has failed puzzlingly in a few other contexts. There is a need to explore the different biases of the ATPR in depth and systematically, and in relation with various study contexts. This project offers the opportunity to do so. To achieve this aim, it will use a mixed-method formative study, the generalized NSUM model, varying points of comparison (self-report, RDS and national estimates), as well internal validation tests involving non-sensitive quantities. The current network-generator question based on secret sharing also still raises some questions worth exploring further. Finally, the level of stigma, which may vary across sites, may also affect ATPR implementation.

The study should also bring light on whether, where and how RDS can be used to collect quantitative data on abortion safety, and whether it is possible to reach equilibrium when constituting samples of abortion seekers. The pre-test is expected to bring much light on this issue; the multiplex RDS provides an interesting avenue to expand on current applications and could be well suited to conducting RDS in cases the practice of interest does not bring individuals directly in contact.

In the reminder of the discussion, we will cover the ethical challenges raised by our study and its limitations.

### Ethical considerations

The ethical challenges involved in RDS on abortion are numerous, a topic which we discuss in-depth in this section. The main benefit of this study is that it will generate information on abortion safety in contexts where access to abortion services is restricted: first, using new network-based approaches, we will measure the degree of abortion safety in the two sites. Second, through this experiment, we hope to fine-tune two alternative network-based approaches, so that population-based information on the safety of abortion can be in the future more easily collected in restrictive contexts. To date, information on abortion safety is lacking in contexts where access to safe abortion services is restricted. Such information is needed at the national, regional and global level to help governments improve the entire spectrum of reproductive health services in their efforts to reduce maternal morbidity and mortality, from improving access to safe abortion services where pertinent, to providing harm reduction programs and quality post abortion care programs for illegal abortions. An additional benefit of the study for women lies in their exposure to a discourse that destigmatizes abortion. This study moreover provides the respondents with access to professional help in case they want to prevent unplanned pregnancies: we will indeed arrange for referral for contraceptive counselling for women who ask it in each site.

We considered the following risks for women who participate in the study. First, women will be asked to disclose a practice that is illegal in many cases. To protect women, we will ensure absolute privacy and confidentiality during the interviews, and collect absolutely no identifying information at any point during the study (see details below). Second, we will ask women to disclose a stigmatized practice in the course of this study; the participation to the study itself could thus be stigmatizing if women's entourage are aware of the object of the study. Therefore, while the objectives and content of the study will be clearly explained to participating women once in a private and confidential place, the study will be presented to all others as a women's health survey. Moreover, the results of the study itself will contribute to de-stigmatize this practice, by showing how common unsafe abortions are. Regarding these two risks (illegality and stigma), we should underline that RDS, one of the network-based approaches applied in this study to obtain information on the safety of abortion, has been applied more than 100 times before for other stigmatizing, illegal and health threatening practices, such as men having sex with men or prostitution in HIV research, or sexual violence and illicit drug use [31–33]. These studies have been successfully completed, which is an indicator of respondents feeling safe and able to respond openly. A final potential risk is that women could feel psychologically distressed during the interviews when evoking their abortions. However, the literature clearly shows that psychological distress is not a usual outcome of abortions; in the opposite, abortions usually bring relief to women who are pregnant at an inappropriate time [45]. Moreover, in this study, we will only collect data on the conditions of the abortions as they relate to their safety; we will not explore the reasons for pregnancy termination or the unintended pregnancy. If the woman appears to the interviewer that she requires additional support or if she herself asks for more information or seeks support, we will arrange for referral to psychological counselling in each site. For each site, there is an established collaboration with the local hospital so that referrals can be made smoothly. As the study will be announced to all except the participants as a "women health" study, the abortion of the women will not be revealed in the referral process.

This study will protect the women participating to the study against the aforementioned risks through a number of measures, which will be identical across the two study sites, except in case of additional requirements by national ethics committees. The measures are the following:

#### Recruiting participants

For the ATPR/self-report survey, a random sample will be drawn from the HDSS sampling frame of residents: this mode of selection will be explained to the potential participant. For participants to in-depth interviews as well as RDS participants, interviewers will first work with a few key informants (for example: professionals in social work or community health workers). They will be asked whether they can locate women who had abortions. These key informants will be trained along RDS procedures, to ensure they approach these women in a confidential manner, explain the survey or in-depth interview, and give them a contact where the women can reach a fieldworker if interested in the study. For this project, we will reach out to key informants connected to varying population groups, from schoolgirls, young people out of school, to married women, young mothers, older reproductive age women and migrant women. Care will also be taken in working only with key-informants with a trusted record of collaboration; the longstanding implementation of the research teams in the study areas ensures that a variety of trustworthy key informants can be mobilized.

#### Interview location

Once contacted by potential seeds or IDI respondent, interviewers will agree with them on a secure place to meet. The interviewer proposes a confidential place. Once the potential respondents come to the secure interview place, interviewers apply the informed consent and screening procedures; in case the woman accepts to participate and meets the eligibility criteria, she will be enrolled. The eligibility criteria for IDI will be age, residence. The eligibility for RDS will be age, residence and having had an abortion in the last 3 years (or being close to somebody who had an abortion for the multiplex RDS). To screen for eligibility for the RDS, a large spectrum of reproductive health behaviours and events will be investigated, so that women who end up not being selected will not know what the study is about. Women who accept to participate in the first RDS interviews ("seeds") will be requested at the end of the interview to propose the study to other potential participants; interested potential participants will contact the study team, who will then apply eligibility and consent procedures. Willing participants will be trained to give out referral coupons to potential participants, to keep track of women who were approached but are not interested to participate and asked to come back for a second interview (to document the recruitment process).

#### Inclusion of adolescents

Since the study population includes individual from 15 years onwards, the project engages adolescents. As adolescents are minor, the standard practice in both Burkina Faso and Kenya is to obtain an assent form for them and consent form from their parents. But according to the fact that this topic is sensitive allowances can be made by local ethics committee to permit the adolescent to give the consent. In the event that these allowances are not obtained, the study will be limited to the 18 to 49 year-olds. Privacy and confidentiality for this group will be reinforced.

#### Informed consent

Informed consent will be sought from all participants (in-depth interviews, ATPR/self-report survey, RDS survey). The consent form will be read to participants and explained. The informed consent form will explain that the study is on the help of network members in case of family or reproductive problems for IDI and ATPR participants and on the safety of abortion for RDS participant. It will be made clear that the woman can stop the study at any time. The benefits (no direct benefit, see compensations below) and potential harm of the study (i.e. talk about private and potentially stressing reproductive events) will be made clear to participants. The measures taken by the study to minimize risks to the participants will be explained: how the study ensures full confidentiality and avoids accidental disclosure will be described. For participants who agree to take part in the study, informed consent will be documented as a verbal consent on a tablet.

#### Compensation

As RDS participants will be asked to go twice to a chosen location to be interviewed, fieldworkers will offer them compensation twice for their transportation costs. Compensations will be site specific. They may be monetary (i.e. reimbursement of transportation costs and/or credits for phone time) depending on local habits and national ethics committees' rules.

#### No identifying information recorded

All women recruited as study participants will be assigned unique ID numbers and no names or addresses will be recorded on the interview tools. There will be no identifying information on the consent form nor on the questionnaires, nor on the audio recording of the in-depth interviews. We will also ask for contact information (phone numbers) and permission to be contacted again for the second RDS interview. At this occasion, the contact information will be written down directly in an encrypted format on a separate form also containing the participant’s ID number, assigned at the same time. The encrypting key will be computer generated, and saved by the fieldworkers on their tablets and then at the University of Geneva in a computer file itself safeguarded on a computer protected by a password.

#### Privacy during interviews

All discussions and interviews will be held in a perfectly private space, with no one else assisting except the interviewer and the participant. For household surveys (ATPR/ self-report tool) and in-depth interviews, the following procedures will be enforced. The interview will only take place if a perfectly private place (like a secluded room, or a secluded spot in the courtyard or near the house) can be secured. If another person walks in the space, the interview is interrupted and starts again only once the person has left. The IDI and RDS interviews will be held in a private space indicated over the phone once the person calls; the place has to meet the same criteria as above. Fieldworkers will have a safe place to offer if the respondent does not know where to meet.

#### Selecting, training and contracting fieldworkers

Fieldworkers will go through a value clarification procedure, involving the collection of data on the level of stigma they attach to abortion at the beginning and at the end of the training. They will be trained to ensure that they will in no way use pressure or coerce women to participate. They will be trained on how to find first RDS participants ("seeds"). Fieldworkers will be trained in screening participants for eligibility criteria in a non-threatening way and in obtaining informed consent. If the allowance has been made by the country team for adolescents to consent for themselves, then this information will be provided to the trainees. Fieldworkers will moreover be trained in maintaining neutrality during the interview and in making women feel safe and comfortable talking about private reproductive issues with them. This will be even more important if adolescents are included in the study. They will be trained to comply with women who want to stop or refuse to answer one question during the interviews. To increase rapport and trust during the interview, all interviewers will be females; they will not live in the communities nor be known in the communities. Interviewers will be required by contract to maintain perfect confidentiality about the information shared with them by participants. Interviewers will be trained in registering absolutely no identifying information along with the data collected, either via tablets for the quantitative data or consent form or on audio records for the qualitative material. They will be trained to explain to participants the use of tablets for data recording. They will also be trained to explain how the anonymity of the data is fully preserved during the study.

#### Quantitative data collection software

Data collection for the ATPR and RDS component of the study will be conducted on handheld android tablets using the mobile data collection software SurveyCTO collect. SurveyCTO is a platform that facilitates offline electronic data collection. Electronic data collection will be used to allow for increased security of collected data; tablets will be password-protected and completed consent forms and questionnaires will be uploaded to a secure server at the end of each day. Interviewers will also carry paper copies of the instruments however, in the case of power or mechanical failure. Paper copies will be entered as soon as tablets are functional again and will be destroyed thereafter either by shredding or through incineration. Interviewers will pick up and return their tablets daily to the HDSS offices where they will be stored in locked cabinets. Only the lead scientist on each site will have access to these cabinets. Interviewers’ passwords will allow them to access the data collection tool but not to see or edit previously entered questionnaires on the tablet. Backups are automatically stored on each tablet in case they were are unable to transmit the data immediately and automatically to the remote server. The backup data will remain on the password-protected tablets until the end of field activities, and until all the data have been synced with the SurveyCTO Server, at which time each tablet will be securely and permanently wiped clean. When not in use, all tablets will be secured in lockable cabinets or containers/cases in the HDSS field offices accessible only to the lead scientist of each site.

#### Data security

Encryption and decryption keys will be generated by security software that comes with the SurveyCTO package and which is automatically available to the project through the study’s SurveyCTO account. The encryption key will be programmed into all questionnaires to ensure that the responses are encrypted prior to transmission to the remote server. The study team will have the private decryption key, which will be saved by the study investigators in a file on a secure computer accessible only to the PIs. Once a questionnaire is completed, all the sensitive data it contains (identification number and nicknames or initial for network numbers, mention of abortions) will be automatically encrypted during the transmission to our secure, private, remote server space within the SurveyCTO Server. Thereafter encrypted data will be downloaded onto computers by the study investigators using a desktop application called SurveyCTO Sync.

Data will be downloaded from the study server servers to cold room computers by the research team or persons appointed by them where they will be decrypted using the private key (during transmission it undergoes double encryption again). Thereafter all personal identifying information will be removed from the dataset before it is made available to other members of the research team for analysis. Although local backups of the data are saved on the computer and servers, just in case of a crash, it remains encrypted and therefore unreadable until the private decryption key is entered. At the end of the study period, all the data on the tablets will be wiped, final data collection tools, datasets and stata do files for importing data will be downloaded from the server and the server wiped and closed.

Decryption and storage of raw data will occur on one secure computer, accessible only to the PIs, for the duration of the study. All decrypted data will be stored on regularly backed up secure drives on password protected computers within the study institutions. Data will only be accessible to members of the study team for analysis. A master data set containing data from all countries will be held centrally at the University of Geneva, with the Geneva research members having access to this data set, and made available to HDSS sites as needed for the central products. Country specific data files will be available to researchers from the HDSS sites for future analyses, beyond the currently promised products. WHO, the University of Geneva and each center running the HDSS sites are the primary owners of each sites study data. All study data will be destroyed after 10 years.

### Limitations of the study

Despite their promise to measure abortion safety in highly restricted contexts, network-based survey approaches will inevitably miss a portion of abortions, those of women who had a direct access to the health system and those who used common-knowledge methods. However, given that information on the safety of abortion comes so far almost exclusively from hospital-based data (women who seek care after a complication, estimated at 6.9 million in 2012 that is 27% of all unsafe abortion globally) [46], the network-based sample of abortions which can be expected through network approaches (about 70–80% of all abortions in highly restrictive settings) represents a great improvement.

Moreover, while a four sites study would have been ideal (one rural and one urban site in two countries), this design is a compromise between the requests for generalization (more than one country) and budgetary constraints. Sub-Saharan Africa was chosen as the region with the highest incidence of least safe abortions.

A last concern is the reliance of the study on key informants for the formative stage and implementation of RDS. Thoughtful and purposive recruitment of these informants is key to the success of this study, since key informants who are too similar are likely to recruit women with overlapping network interactions and some parts of the networks will be missed. We outlined above the various steps taken to limit risks in this regard: the choice of Health and Demographic Surveillance Systems, whose researchers know the study populations, and the high capacity of the staff involved in fieldwork.

## Data Availability

Data generated through this project will be available upon request; it will be archived for public use according to WHO procedures.

## Abbreviations

APHRC: African Population Health Research Center
ATPR: Anonymous Third Party Reporting
HDSS: Health and Demographic Surveillance Systems
HRP: Human Reproduction Programme
HRPLID-HUBS: Human Reproduction Programme Long term Institutional Development Hubs
IRSS: Institut de Recherche en Sciences de la Santé
IDI: In-depth interviews
NSUM: Network Scale-Up Method
RDS: Respondent-Driven Sampling
WHO: World Health Organization

## Footnotes

1 Hidden populations were defined in 1997 by Douglas Heckathorn as populations for which no sampling frame exists so that their size and boundaries are unknown, a definition which includes groups whose members engage in a given stigmatized or illegal behaviour and do not wish to disclose it [10].

2 From here on, "abortion safety" is used as a synonym for abortion care seeking characteristics (method, provider, setting) and its consequences (symptoms and post abortion care-seeking).

3 The first experiments with the ATPR have used the following definition of close relations: "women who share secrets with you". Updates use a reciprocal question (women with whom you share secrets and vice versa" [11–19]. The “best friend" definition [22] is reciprocal and possibly less biased towards women who had an abortion as "secrets" may direct women to abortion; at the same time, asking only about 1–3 best friends unnecessarily limits the data that can be collected both regarding the number and type of close relations. In this study, we will test a question to generate the network of close relations validated in international surveys: "whom do you discuss important matters with" [23–25]; we will test if limiting numbers improve estimates.

4 In a number of countries with liberal laws but insufficient provision of legal abortion services (India, South Africa, Ethiopia, Nepal, Bangladesh), only some abortion seekers need to disclose to network members; network-based methodologies will in such settings document only part of the abortions and yield non-representative data [40].

5 Our simulations show that a multiplex RDS does not perform worse than a classic RDS in terms of the estimates it produces at the end.

6 Previous application of the ATPR with a sample of men showed that they report on the abortions of their close female relations, but that reports are slightly less numerous and biased towards complicated cases [11].
